# Effects of Red-Backed Salamanders on Ecosystem Functions

**DOI:** 10.1371/journal.pone.0086854

**Published:** 2014-01-22

**Authors:** Daniel J. Hocking, Kimberly J. Babbitt

**Affiliations:** Department of Natural Resources and the Environment, University of New Hampshire, Durham, New Hampshire, United States of America; Universität Zurich, Switzerland

## Abstract

Ecosystems provide a vast array of services for human societies, but understanding how various organisms contribute to the functions that maintain these services remains an important ecological challenge. Predators can affect ecosystem functions through a combination of top-down trophic cascades and bottom-up effects on nutrient dynamics. As the most abundant vertebrate predator in many eastern US forests, woodland salamanders (*Plethodon spp.*) likely affect ecosystems functions. We examined the effects of red-backed salamanders (*Plethodon cinereus*) on a variety of forest ecosystem functions using a combined approach of large-scale salamander removals (314-m^2^ plots) and small-scale enclosures (2 m^2^) where we explicitly manipulated salamander density (0, 0.5, 1, 2, 4 m^−2^). In these experiments, we measured the rates of litter and wood decomposition, potential nitrogen mineralization and nitrification rates, acorn germination, and foliar insect damage on red oak seedlings. Across both experimental venues, we found no significant effect of red-backed salamanders on any of the ecosystem functions. We also found no effect of salamanders on intraguild predator abundance (carabid beetles, centipedes, spiders). Our study adds to the already conflicting evidence on effects of red-backed salamander and other amphibians on terrestrial ecosystem functions. It appears likely that the impact of terrestrial amphibians on ecosystem functions is context dependent. Future research would benefit from explicitly examining terrestrial amphibian effects on ecosystem functions under a variety of environmental conditions and in different forest types.

## Introduction

Ecosystems supply critical services for human societies including food, clean air, and potable water. These services are supported by a variety of ecosystem functions such as primary production, nutrient cycling, and soil formation [1], [2]. Despite the importance of these functions in supporting ecosystem services, it remains difficult to predict how organisms contribute to specific ecosystem functions. Predators can affect ecosystem functions indirectly through top-down trophic cascades and directly by altering bottom-up nutrient dynamics (e.g. excretion of soluble nitrogen).

Much of our current understanding of the effects of predators on terrestrial ecosystem function comes from examination of trophic cascades. Predators are able to affect primary producers indirectly through predation on herbivores. In terrestrial ecosystems, carnivores generally reduce herbivore abundance, thereby reducing herbivore damage on plants and increasing plant biomass and reproductive output [3]. However, these patterns are not consistent across all predator species and habitats. No clear pattern has emerged to predict when predators increase or decrease primary production, but it may be related to intraguild predation [4], foraging strategies [5], behaviorally-mediated indirect interactions [6], and the balance of top-down and bottom-up effects [7]–[9]. Additionally, plants processing anti-herbivore defenses (including ant-tending) and systems with high herbivore diversity attenuate top-down effects on primary producers [3].

**Figure 1:**
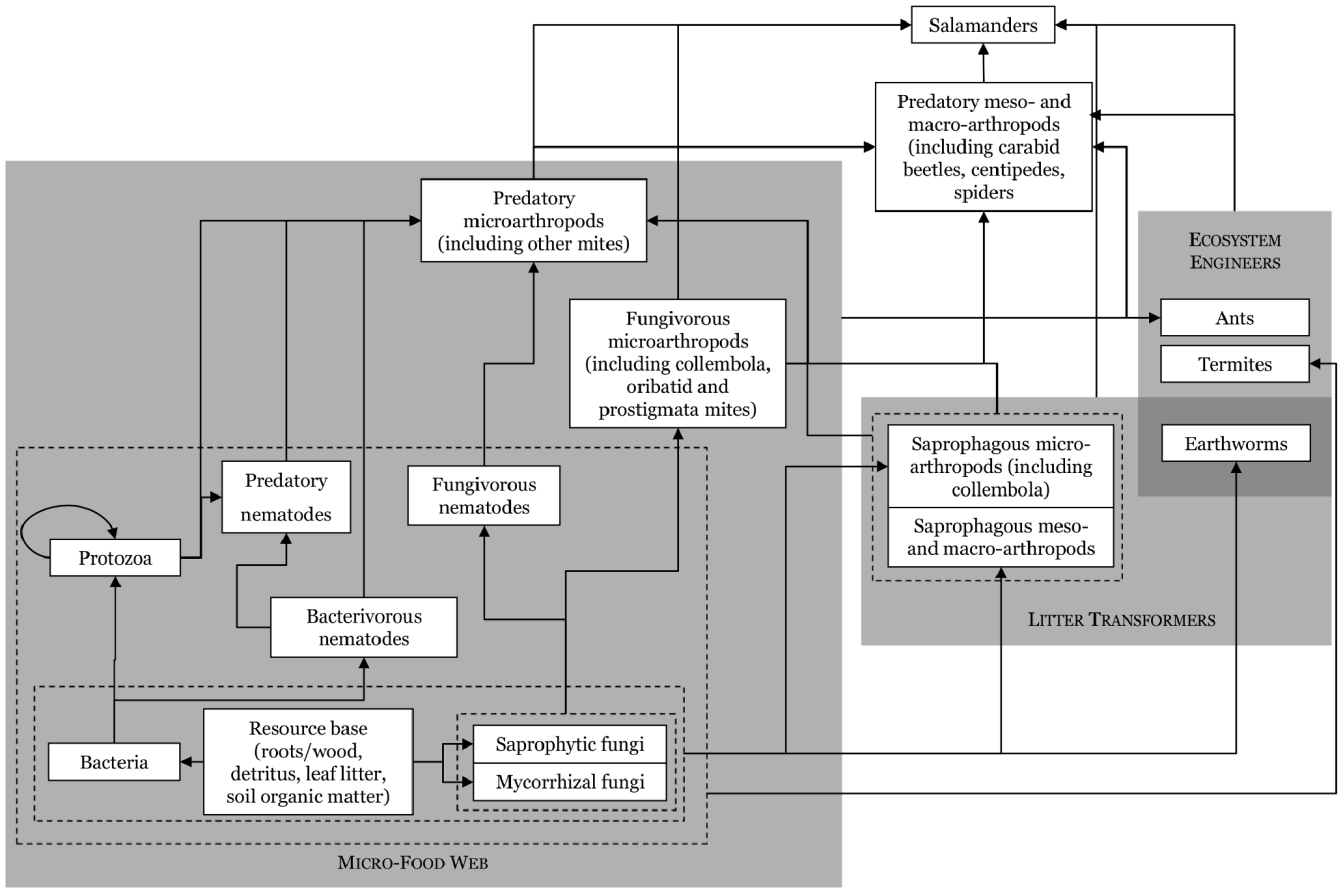
Soil-litter food web including red-backed salamanders as one of the top predators in the system. All organisms in the system contribute nutrients through waste excretion back into the resource base (not shown). The effect of salamanders on ecosystem functions may be a balance of complex trophic cascades through direct predatory effects, indirect behavioral responses of prey to predators, moderated behavior of predators in response to intraguild predators, or changes in nutrient dynamics associated with changes in the food web. Adapted from Coleman and Wall [58] figure 7.2.

Studies in terrestrial systems examining ecosystem functions other than primary production have been even more conflicting. Some predators reduce nutrient availability [5], [10], while others increase nutrient availability [5], [7], [11]. Terrestrial amphibians can increase, decrease, or have no effect on litter decomposition rates depending on the species and habitat [8], [12], [13]. These idiosyncratic results may arise through differences in predator diversity, functional redundancy of herbivores, indirect effects on behavior or anti-herbivore defenses, or initial productivity and nutrient pools [5], [14]. Differences may also arise due to experimental venue with small, controlled experiments often revealing processes not detected in larger field manipulations [15]–[17]. Variable results may also arise due to the complexity of the detrital food web and the diverse prey consumed by many amphibians. For example, a salamander may feed on predaceous mites that feed on fungivorous collembola, thereby indirectly increasing collembolan abundance, but the salamanders may also prey directly on collembola, directly offsetting the effects of preying on mites (Fig. 1). The balance of these feeding pathways would influence saprotrophic fungi and therefore leaf litter decomposition.

Predators can also affect ecosystem functions by altering nutrient dynamics. Predators ingest energy and nutrients through consumption of prey. Some of these ingested resources are used to produce new tissue and the rest is passed back to the environment through dead tissue, excreted waste, heat, and respiration [9]. The excretion of waste by vertebrate predators often provides nutrients in forms readily used by microbes and primary producers and can increase decomposition and plant growth in some systems [7], [11] but see [8]. These bottom-up effects on nutrient dynamics may have complementary or opposing effects on particular ecosystem functions when paired with top-down trophic cascades.

To better understand the effects of predators on ecosystem functions, we experimentally manipulated the density of red-backed salamanders (*Plethodon cinereus*), one of the most abundant vertebrate predators in many eastern forests [18]. Lungless salamanders of the family Plethodontidae can occur at densities of 0.2–8.2 m^−2^ [19], [20] and can have a biomass twice that of all the passerine birds and equal to all the small mammals in a forest [21]. This tremendous biomass has led many ecologists to speculate on the importance of salamanders in ecosystem functions [22], [23]. As vertebrate predators with efficient conversion of food into tissue, woodland salamanders may be important contributors to ecosystem function. Woodland salamanders may influence litter decomposition and potentially net primary production (NPP) [13], [23] through their role as abundant predators in the detrital food web [21]. Hairston [23] estimated that, at average densities in the southern Appalachians, 1.165 kcal per m^2^ of energy is stored in red-backed salamander biomass, a caloric content greater than all other vertebrate predators combined. He also estimated that salamanders consume more than a complete turnover of the soil invertebrate fauna each year [23]. Additionally, control of herbivorous and leaf-fragmenting invertebrates could alter nitrogen availability and plant growth.

To date, studies on the effect of red-backed salamanders on ecosystem functions have primarily been limited to litter decomposition and remain equivocal [13], [24]. We examined the effects of red-backed salamander removal on a variety of ecosystem functions and on a spatial scale an order of magnitude larger than their home range. We conducted this study over 4 years to provide information on the effects of predator removal on the system beyond just the initial perturbation. We also used mesocosm enclosures to more finely manipulate vertebrate predator density in a more controlled experiment. Based on previous research and potential effects on the food web and nutrient dynamics, we expected salamanders to 1) reduce the rate of litter decomposition through predation of litter fragmenting invertebrates [13], 2) increase wood decomposition rates through consumption of fungivorous collembola [18], 3) increase nitrogen immobilization by excretion of waste in soluble forms and increased fungal productivity [7], 4) reduce potential nitrogen mineralization rates due to increased needs of fungi, 5) reduce nitrification rates in response to fungal productivity, 6) increase acorn germination through consumption of herbivorous invertebrates [18], 7) reduce foliar insect damage on red oak seedlings, and 8) reduce the abundance of intraguild predators such as spiders, centipedes, and carabid beetles, primarily through competition [25], [26].

## Materials and Methods

### Ethics Statement

We took measures to reduce handling time and distress of all vertebrate animals in this study. All research was conducted with University of New Hampshire IACUC approval 080301, 091106, and 110403.

### Experiment 1: Effects of Salamander Depletion

In April 2008, we established ten 20-m diameter circular plots (314 m^2^) in American beech (*Fagus grandifolia*) dominated forest stands at the University of New Hampshire’s Kingman Farm property (146 ha.). Plots of this size are approximately 13 times the size of a red-backed salamander home range [27] and similar to the size of depletion plots previously used to study woodland salamander competition [23], [28]. We randomly assigned half the plots (*n = *5) for salamander depletion and the assigned remaining as reference plots (*n* = 5). Plots were all located within 1 km of each other and separated by a minimum of 20 m.

In April - November, we conducted visual encounter surveys (VES) for salamanders on 91 nights from 2008–2011, primarily during or following rain events to remove salamanders from plots and establish experimental treatments. We sampled in all months but more frequently in spring and fall on rainy nights when salamanders were most active. Based on weather conditions, we sampled at infrequent intervals from daily to weeks between surveys. To avoid correlation between plots, we randomly selected a starting plot each night and then proceeded to subsequent plots in the most convenient order, which differed depending on the starting plot. Survey routes within each plot followed concentric rings marked with twine so that an entire plot was covered only once each night. In reference plots, we counted individual red-backed salamanders but did not disturb them. One to four researchers walked the plots at sufficient pace to avoid double counting of wandering salamanders during a survey night (20–30 person-minutes per plot). Survey methods were the same in reference and depletion with the exception that in depletion plots any encountered individual was collected by hand and brought back to the laboratory and euthanized using a 1% solution of MS-222 and preserved in ethanol for potential use in research.

To minimize immigration and edge effects, we delineated a 12 m diameter central “core” of each plot, creating a 4 m wide buffer where we removed salamanders but did not measure ecosystem functions. We flagged the location of each removed salamander and later recorded the distance from the plot center. This provided a way to examine if a greater number of individuals were caught near the plot edge, indicating immigrants from outside the plot were being caught as they entered the plot [29]. We did not conduct intensive mark-recapture estimates on our plots because it would have been impossible to conduct sufficient removals while doing mark-recapture. Additionally, red-backed salamanders are highly fossorial resulting in low detection probabilities, which leads to extremely large uncertainty in population estimates [30]–[32]. Therefore, to get any reliable population estimates would require the addition of coverboards, fencing, and litter searches [30]–[32], which would alter ecosystem processes and confound our metrics of interest.

#### Decomposition

We measured litter decomposition rates using two methods. We used bags (20×20 cm) constructed of fiberglass window screen with 2-mm mesh and filled with 10.2 g of air-dried (to constant mass) beech leaf litter [33] then sewn shut, and we used 1-m^2^ leaf litterboxes with larger (1 cm) mesh openings on the top and bottom, and filled with 255 g of air-dried litter. Boxes were surrounded by landscape edging to prevent leaves from blowing out and were staked down to secure the box and leaves. This quantity of leaves used is in the range of annual deciduous leaf fall in the region [33]. We collected freshly fallen leaf litter in late October to early November each year and air dried it in the laboratory for more than a week [33]. Litterbags and boxes were filled and placed in the field at the beginning of each December using a stratified pattern from the same pool of dried leaves to avoid bias when comparing the methods. Three litterboxes and nine litterbags were randomly located within each plot core. We collected one random litterbox and three litterbags from each plot after 6, 12, and 18 months, with the exception of the final year when we only measured decomposition at 6 and 12 months. We then oven-dried the leaves at 60°C to examine the mass lost over the time period. We corrected the initial mass for the difference between air and oven drying [33].

Using both litterbags and litterboxes is important to determine how salamanders affect decomposition of fine litter because the smaller mesh bags exclude many invertebrates. These two methods could help explain previous conflicting results [13], [24], [35], and test competing hypotheses that salamanders primarily affect decomposition through consumption of large, leaf-fragmenting invertebrates [13] or through consumption of fungivorous collembola [23].

We also examined the effect of red-backed salamanders on woody decomposition. Litter is fragmented by a variety of invertebrates and decomposed by both bacteria and fungi, whereas wood decomposition is almost entirely driven by fungi [33]. Salamanders could affect saprotrophic fungi through predation on collembola, which feed on fungi, allowing us to further test competing hypotheses [13], [23]. We used untreated birch dowels (6.35 mm diameter x 30 cm long) to measure woody decomposition. We enclosed each dowel in 2 mm fiberglass mesh sleeves, to enable extraction, and hammered them vertically 20 cm into the ground so that 10 cm of each dowel was above the soil surface [33]. Dowels were placed 10–20 cm from litterbags and were collected at the same time as litterbags and boxes. To determine mass loss over time, we weighed each air-dried dowel and attached a uniquely numbered aluminum tag prior to installation in the field. We oven-dried every tenth dowel to determine a correction for the difference between air and oven drying but did not use these oven-dried dowels in our study as they may have altered hydrophobic properties. Upon collection from the field, we oven-dried the dowels at 60°C and carefully removed any soil and attached fungal hyphae before weighing to determine mass loss.

#### N-mineralization rate

We used laboratory incubations to measure potential nitrogen mineralization and nitrification rates. In fall 2009, 2010, and 2011 we collected the organic layer from six random locations within each plot. We measured inorganic nitrogen levels from each location immediately and incubated the remaining soil in thin-walled polyethylene bags at a constant temperature (25°C) and humidity (50%) for 28 days [36]. We extracted inorganic nitrogen using 2M KCl, then filtered and froze samples at −20°C until analysis. We measured nitrate and ammonium using an Astoria autoanalyzer [Astoria-Pacific International, Clackamas, OR; 37,38], where ammonium was quantified using the indophenol-blue method [37] and nitrate was quantified using the vanadium (III) reduction color reaction modified for microplate assays [38]. We calculated net nitrogen mineralization and nitrification rates over 28 days from the difference between the initial and incubated samples [36].

#### Oak germination

We planted red oak acorns in each plot to determine the effect of salamanders on germination rates through the consumption of herbivorous invertebrates or reduced feeding activity of herbivores in response to the presence of a predator [39], [40]. We collected freshly fallen acorns in early autumn of 2008–2010 and stored them in moist conditions at 4°C over the winter. In April, we planted 20 acorns per plot under the leaf litter in 2009 and 2010 and 40 acorns per plot in 2011, on the soil surface [41]. We covered the acorns with mesh cages to prevent disturbance from vertebrates. We checked for germination weekly throughout the growing season to record the total number of acorns germinated.

#### Litter-dwelling macro-invertebrate predators

The densities of litter-dwelling predaceous invertebrates may also affect ecosystem functions compounding or mitigating salamander effects. Therefore, we quantified the abundance of three major macro-invertebrate predator groups: adult carabid beetles, centipedes, and spiders. We collected 0.5 m^2^ leaf litter from a random location within the central core of each plot in the spring, summer, and fall of each year. We extracted invertebrates from the litter using large Berlese funnels and enumerated the three predator groups [42].

#### Statistical analysis

We employed a repeated measures multivariate analysis of variance (rmMANOVA) to test the effect of salamander depletion on ecosystem functions. We used mean decomposition rates for each year, arcsine-transformed proportion of acorn germination, potential nitrogen mineralization rate, and potential nitrification rate as the multivariate response. We used treatment as the primary effect with repeated measures for each of 3 years 2009–2011. Analysis was conducted using the “Manova” function from the car package [43] in R [44].

### Experiment 2: Effects of Salamander Density on Ecosystem Functions

Conducting large-scale experiments has the benefit of high realism and a potentially broader scope of inference compared with small-scale experiments, but lacks the precision and control of small-scale experiments. Therefore, we also conducted a smaller scale enclosure experiment to more finely manipulate red-backed salamander density. In May 2010, we constructed 20 mesocosm enclosures (1.41 m × 1.41 m × 1.00 m tall). The mesocosms were enclosed in aluminum (sides) and fiberglass (top and bottom) window screen (2-mm grid) with a secure window screen lid. All enclosures were located within a 40-m radius, in a forest stand dominated by American beech on UNH’s Kingman Farm property, within 50 m of two plots from experiment 1. We buried the lower 30 cm of each enclosure belowground. We carefully removed the soil in blocks and replaced it inside the enclosure on top of the mesh screen then added the leaf litter back on top of the soil (following Wyman [13]). Immediately upon addition of the leaf litter, we searched it to ensure no salamanders were added back into the enclosure. During 2010, we allowed soil to settle, fine roots and fungal hyphae to reestablish, and microarthropods and flying insects to recolonize. We left the mesh lids open until April 2011 to allow insect recolonization, while 10 cm window screen baffles overhanging over the top of the enclosure prevented recolonization by salamanders. We added a single hemlock coverboard (1 m x 20 cm x 5 cm) to each enclosure to serve as refuge for salamanders. During 2010 and spring 2011, we conducted nocturnal visual encounter surveys and daytime coverboard searches to remove any salamanders that may have entered during soil or litter replacement.

On 01 May 2011, we collected red-backed salamanders from the forest within 1 km of the enclosures and brought to them the laboratory. All salamanders used in this experiment were adult males or adult, non-gravid females as verified by candling [45]. Within 48 hours of capture, we haphazardly put salamanders in containers one at a time in a stratified pattern until each container had 0, 1, 2, 4, or 8 salamanders. Salamanders were then anesthetized in a 1% solution of MS-222 [46] and given one of eight marks using VIE such that each salamander within an enclosure had a unique mark. Marking was intended to allow for identification of intruders into the enclosures. We then randomly assigned each container to an enclosure, which resulted in salamander density treatments of 0, 0.5, 1, 2, or 4 salamanders per m^2^. This range of densities covers the natural variation in red-backed salamander density [18], [27] and resulted in four replicates of five treatments.

In enclosures, we measured the same metrics of ecosystem function as described for experiment 1. To accomplish this, we added six litterbags, one litterbox, six birch dowels, and planted 20 red oak acorns in each enclosure. Litterbags and litterboxes were added to the enclosures in December 2010 to coincide with natural beech litterfall. We added the dowels in April 2011. To ensure enclosures were devoid of salamanders at the start of the experiment, we checked the coverboards weekly in April and again when the salamanders were added to the enclosures. We also checked for surface-active salamanders on five rainy nights in April. Any salamanders found were removed and released on the outside of the enclosure. We then added marked salamanders to each enclosure on 03 May 2011. We also checked under the coverboards every 7–10 days during the experiment to check for unmarked individuals. Only one small, juvenile was found unmarked in an enclosure at the beginning of June and was promptly removed. Each week we also recorded the number of newly germinated acorns and marked them with a small zip tie.

In September 2011, we removed all germinated acorn seedlings and measured total leaf area and foliar insect damage using WinFolia (Regent Instruments, v2009a). In addition, we collected soil samples from the organic layer to examine potential nitrogen mineralization rates in October 2011. We used the same techniques to measure ecosystem functions as described in experiment 1 with the exception of litterbag and wooden dowel decomposition. We collected 1 litterbag and 1 dowel from each enclosure monthly beginning in April 2011. From this repeated sampling we were able to calculate the rate of decay using the equation

where *M* is the mass remaining at time *t*, *M_o_* is the initial mass, and *k* is the decay constant.

We also quantified the abundance of macro-invertebrate predators (adult carabid beetles, centipedes, and spiders) at the end of the study. We extracted invertebrates from the litterbox litter using large Berlese funnels and enumerated the three predator groups. Finally, we used coverboard and nighttime visual encounter searches in September – November 2011 to remove salamanders and quantify survival (final density).

We analyzed the effect of salamander density on ecosystem functions using MANOVA. Additionally, we were interested in the potential influence of salamander survival, invertebrate predator abundance, and inorganic nitrogen pools in conjunction with initial salamander density on ecosystem functions. Therefore, we performed Multivariate Analysis of Covariance (MANCOVA) with the addition of salamander final density, amounts of nitrate and ammonium at the start of the incubations, and total abundance of invertebrate predators. For significant multivariate analyses, we used univariate linear regressions to determine the direction and magnitude of effect on each of the ecosystem functions. Analysis was performed in R [44] using Manova in the car package [43].

We analyzed the foliar insect damage separately from the other ecosystem functions because 3 enclosures had zero acorns germinate. Therefore, proportion of foliar insect damage could not be calculated and we did not want to use this reduced sample size for the analysis of all ecosystem functions. We used a linear regression to examine the effect of salamander density on foliar insect damage.

## Results

### Experiment 1: Effects of Salamander Depletion

**Figure 2:**
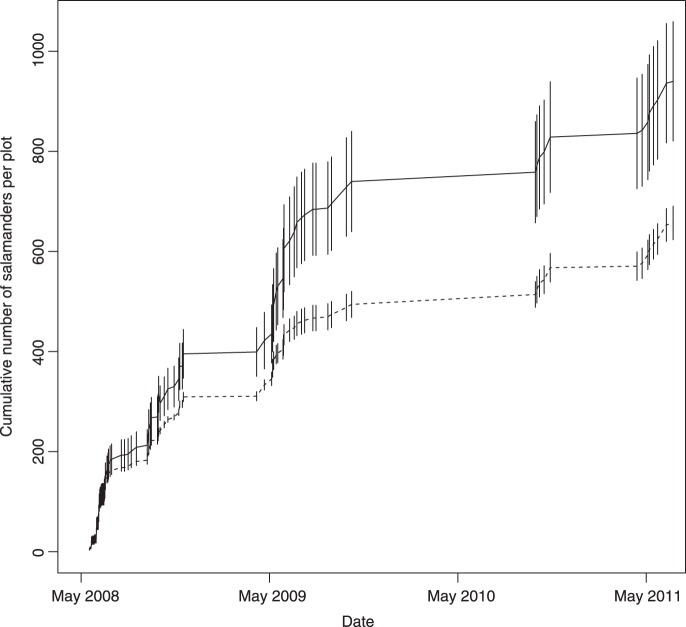
Mean cumulative number of captures per plot (±1 SD) observed in the reference plots (solid line) and removed in the depletion plots (dashed line).

We removed red-backed salamanders from all depletion plots on 91 nights from 2008–2011. This resulted in the removal of 3,309 individuals from the five depletion plots (662±32 individuals per plot), an average reduction of 2.1±0.1 salamanders per m^2^. This compares with a total of 4,645 salamanders observed on the same occasions on the reference plots (∼29% reduction in depletion plots). As we surveyed plots repeatedly, the cumulative number of salamanders observed increased at a greater rate over time in the reference plots compared with the depletion plots (Fig. 1). Although this doesn’t elucidate the magnitude of the difference in abundance among treatments, it does suggest that there were fewer salamanders to observe on the depletion plots. The number of salamanders observed per night averaged over each month was consistently greater in the reference plots compared with the depletion plots (Fig. 2).

Despite the 4-m buffer zone around each plot, we were concerned about immigration from surrounding habitat. Using visual implant elastomer, we marked 124 individuals in the 2 meters beyond the edge of the buffer zone (10–12 m from center of the plot) around 2 depletion plots from 09 May –08 July 2009 [47], [48]. We subsequently captured 6 of these individuals in the buffer zone and only 1 salamander was found in the plot beyond the buffer zone. This individual was captured 66 cm into the plot past the buffer. Further, the locations of removed salamanders were almost evenly dispersed with respect to distance from the center of each plot. The distribution of salamanders around the plot was fit with a beta distribution as a function of distance from the plot center, accounting for the increasing area with increasing distance from the center (radius^2^). The bootstrapped 95% confidence intervals of the beta distribution parameters overlapped or were very near 1, indicating a near uniform distribution of salamanders throughout the plot (Table 1). Therefore, there is no evidence that we caught more salamanders near the outer edge of the plots or that we had significant immigration into the plots.

**Table 1:** Shape parameters defining the estimated beta distributions of salamander removals from the center of the plots.

	Shape 1			Shape 2		
	0.025	Estimate	0.975	0.025	Estimate	0.975
AllRemovals	0.98	1.09	1.14	1.02	1.11	1.16
2008	1.02	1.16	1.30	1.06	1.22	1.37
2009	0.95	1.04	1.14	0.97	1.05	1.14
2011	0.98	1.15	1.33	0.99	1.18	1.37

Beta [1,1] indicates the density of salamanders removed is uniform with respect to distance from the plot center. The 95% confidence intervals were estimated from 1000 bootstrap iterations. Salamanders were not removed in 2010.

The means of each ecosystem function and mean density of macroinvertebrate litter predators are summarized in Table 2. Nitrogen mineralization rates were generally low and there was virtually no potential nitrification in fall soil samples from any plot. Red oak acorn germination rates were high in 2009 but very low in 2010 and 2011. Mass loss from litterbags was higher than from litterboxes and was similar to rates of woody mass loss from birch dowels (Table 2). Although dominated by small, juvenile spiderlings, spiders were by far the most abundant of the litter predators followed by centipedes (Table 2).

**Table 2:** Summary of means and standard errors (SE) for ecosystem functions and predator densities for 10 plots over three years across treatments.

	Experiment 1: Plots						Experiment 2: Enclosures	
	2009		2010		2011		2011	
Function and Predator Abundance	Mean	SE	Mean	SE	Mean	SE	Mean	SE
Nitrogen Mineralization Rate	0.268	0.043	−0.245	0.091	0.825	0.109	0.980	0.071
Nitrification Rate	−0.002	0.003	0.008	0.003	−0.001	0.001	−0.020	0.014
Proportion acorns germinated	0.213	0.048	0.085	0.042	0.010	0.006	0.443	0.072
Litterbag Decomposition Rate (g g^−1^yr^−1^)	0.302	0.011	0.190	0.013	0.263	0.013	0.524	0.035
Litterbox Decomposition (g g^−1^ yr^−1^)	0.125	0.028	0.176	0.038	0.252	0.028	0.392	0.011
Wood Decomposition (g g^−1^ yr^−1^)	0.353	0.038	0.237	0.040	0.144	0.020	0.867	0.091
Proportion Foliar Insect Damage							0.022	0.004
Carabid Beetles (m^−2^)	1.7	0.3	0.3	0.1	1.6	0.5	0.1	0.1
Centipedes (m^−2^)	4.9	0.9	7.8	2.4	8.8	1.9	2.1	0.4
Spiders (m^−2^)	26.9	3.3	52.5	12.3	62.7	11.0	173.5	16.3

Data are from American beech stands in a New Hampshire forest where half the plots had reduced red-backed salamander abundance and from enclosures in similar stands with densities of salamanders from 0–4/m^2^.

There was no significant effect of treatment or treatment by year, but there were significant differences among years on ecosystem functions (Table 3). Nitrogen mineralization rates were lower in 2010 and higher in 2011 compared with 2009 (Table 2). There was also a lower rate of decomposition in the litterbags in 2010 than in 2009, while woody decomposition was lowest in 2011 (Table 2). There was also a significant effect of year on invertebrate predator abundance with fewer carabid beetles in 2010. There was no effect of treatment or treatment by year on predator abundances (Table 3).

**Table 3:** Results of repeated measures MANOVA.

Type II Repeated Measures MANOVA Tests: Pillai test statistic					
Ecosystem Functions					
Factor	Pillai	approx F	num df	den df	P
Treatment	0.00276	0.022	1	8	0.8854
Year	0.82665	16.691	2	7	0.0022
Treatment*Year	0.17951	0.766	2	7	0.5003
**Invertebrate Predators**					
** Factor**	**Pillai**	**approx F**	**num df**	**den df**	**P**
Treatment	0.27791	3.079	1	8	0.1174
Year	0.58595	4.953	2	7	0.0457
Treatment*Year	0.15638	0.649	2	7	0.5515

Treatment tests the difference between red-backed salamander depletion and reference plots. The ecosystem functions nitrogen mineralization, nitrification, acorn germination, and decomposition rates of litterbags, litterboxes, and wood dowels were repeatedly measured in three years 2009–2011. The effect of treatment on the densities of spiders, centipedes, and carabid beetles was also tested using the Pillai test statistic.

### Experiment 2: Enclosures

During a total of 19 surveys at the end of the study (01 September –24 October 2011), we captured 40 of the initial 60 animals stocked in the enclosures. Only two enclosures had more individuals than initially stocked. One plot was stocked with zero salamanders but one individual was found. The other was stocked with two but five were found. This anomaly was likely due to a wide, thin, ground-level rip in the windowscreen mesh that occurred during a storm (Hurricane Irene) on 28 August 2011. The rip was missed when checked on 30 August and was not repaired until 03 September. Immigration at this late stage of the study would have been unlikely to influence the ecosystem function metrics. There were likely additional animals that survived in the enclosures but were not captured during these surveys because they remained underground. It is unlikely that many if any salamander escaped except at the end in the one ripped enclosure because they were fully sealed with 1-mm mesh windowscreen on all 6 sides and stapled and glued at all edges except the top, which was secured with 2.5 cm wide strips of industrial strength Velcro.

A total of 177 acorns germinated, a mean (± SE) of 44.25 (±7.2)% germination per enclosure. The mean decomposition rate was 0.524 (±0.035) g g^−1^ year^−1^ in the litterbags and a mean of 39.2% mass was lost from the litterboxes, a mean rate of 0.392 (0.011) g g^−1^ year^−1^. Mean potential nitrogen mineralization was 0.980 (0.071) µg N g^−1^ dry soil day^−1^ and net nitrification was −0.020 (0.014) µg N g^−1^ dry soil day^−1^. Of the acorns that germinated, the mean leaf area produced was 690 cm^2^ per enclosure (17.8 cm^2^ per leaf and 89.7 cm^2^ per plant), while the mean proportion of foliar insect damage was 2.2% of the total leaf area produced (Table 2). Of the approximately 175 predaceous invertebrates in the enclosures, 99% were spiders and 93.3% of the spiders were small, mostly hatchlings <3 mm in length (Table 2).

There was no significant effect of salamander density on the ecosystem functions measured (Table 4). We then tested if controlling for the final number of salamanders captured as a covariate in a MANCOVA resulted in a significant effect on ecosystem functions but it did not (Table 4). We performed another MANCOVA to test for the effect when other litter predator density (carabid beetles, centipedes, and spiders) and initial nitrate levels (g g^−1^ dry soil) before incubation were included as covariates. The density of macro-invertebrate predators did not have a significant affect, but initial nitrate levels significantly affected ecosystem functions. However, controlling for these effects did not result in a significant effect of salamander density on ecosystem function (Table 4). Post hoc univariate tests revealed that the significant effect of fall nitrate level on ecosystem functions was driven by its effect on nitrification rates. There was a significant negative effect of initial nitrate levels on potential nitrification rate (t = −503.15; df = 1, 16; P<0.0001). We did not find any significant effect of salamander density, final salamander density, or density of invertebrate predators on the proportion of foliar insect damage on red oak seedlings (Table 4). The estimated magnitude of the effects of each variable can be found in Table 5.

**Table 4:** The results of three MANOVAs testing the effects of red-backed salamander density, final capture density, soil nitrate levels (g nitrate per g dry soil) on the ecosystem functions: N mineralization rate, Nitrification rate, proportion acorn germination, litterbag decomposition, litterbox decomposition, and woody decomposition.

Type II MANOVA Tests: Pillai test statistic					
Ecosystem Functions					
Factor	Pillai	approx F	numdf	dendf	P
Density	0.342	1.127	6	13	1.0000
Density	0.323	0.954	6	12	1.0000
Final Density	0.552	2.465	6	12	0.5191
Density	0.373	1.100	6	11	1.0000
Predator Density	0.660	3.600	6	11	0.1978
Soil Nitrate (g g^−1^)	1.000	31499	6	11	<0.0001
**Linear Regression**					
**Foliar Insect Damage**					
** Factor**	**Estimate**	**SE**	**t**	**P**	
Intercept	0.0223	0.0052	4.3140	0.0037	
Density	−0.0005	0.0023	−0.2060	1.0000	
Intercept	0.0174	0.0051	3.3760	0.0271	
Density	−0.0049	0.0029	−1.6950	0.6735	
Final Density	0.0112	0.0051	2.1950	0.2733	
Intercept	0.0331	0.0126	2.6210	0.1272	
Density	−0.0021	0.0035	−0.5890	1.0000	
Final Density	0.0055	0.0065	0.8580	1.0000	
Predator Density	−0.0001	0.0001	−1.3550	1.0000	

Additionally, linear regression results testing the effect of salamander and invertebrate predator densities on proportion of foliar insect damage on red oak seedlings (arcsine transformed). P-values are Bonferroni corrected for multiple comparisons.

**Table 5:** The relative effects of salamanders on ecosystem functions estimated from MANOVAs.

	2009		2010		2011		2011: Enclosures	
Metric	Estimate	SE	Estimate	SE	Estimate	SE	Estimate	SE
Nitrogen Mineralization Rate	0.070	0.088	−0.018	0.192	0.178	0.223	0.057	0.050
Nitrification Rate	−0.002	0.006	0.003	0.007	0.001	0.002	0.013	0.010
Proportion acorns germinated	−0.175	0.081	−0.110	0.081	−0.010	0.011	0.110	0.059
Litterbag Decomposition (g g^−1^yr^−1^)	−0.027	0.021	0.006	0.027	−0.014	0.027	0.013	0.025
Litterbox Decomposition (g g^−1^ yr^−1^)	0.040	0.059	−0.033	0.079	0.052	0.056	−0.005	0.009
Wood Decomposition (g g^−1^ yr^−1^)	−0.067	0.077	0.023	0.085	0.014	0.043	−0.071	0.064
Proportion Foliar Insect Damage							−0.0005	0.002

The estimates for the experimental plots are the effects of salamander depletion relative to the reference plots. The effects in the enclosure experiment represent the change in the ecosystem function with an increase in one salamander per m^2^. None of the effects presented are statistically significant (P>0.05).

## Discussion

We did not observe any effects of red-backed salamander depletion or density on ecosystem functions. Although researchers have predicted that woodland salamanders are important regulators of ecosystem functions [22], [23], we found no evidence that red-backed salamanders affect litter or wood decomposition, nitrogen cycling, acorn germination, herbivory, or the abundance of other litter predators. This is consistent with Homyack et al. [24] who did not find any effect of red-backed salamanders on oak or maple litter decomposition in a Virginia mixed-hardwood forest, but in contrast to Wyman [13] who showed that red-backed salamanders lowered beech-dominated leaf litter decomposition rates by 11–17%. Homyack et al. [49] suggested the conflicting result with Wyman [13] may have been due to differences in litter type. However, we used American beech litter in both of our experiments, similar to Wyman [13], and did not observe an effect of salamanders. Other studies with amphibians have also found conflicting effects on litter decomposition even when nutrient pools were affected [7], [8], [11], [12]. It is likely that the effect of predatory amphibians on terrestrial ecosystem functions is influenced by a variety of biotic and abiotic factors, giving the appearance of idiosyncratic effects when limited to only a few studies.

Some of the variability in results may be due to the complex nature of litter decomposition. Litter decomposition can be influenced by temperature, moisture, microbial community structure, invertebrate community structure, and available nutrient pools; therefore, the effects of amphibians on litter decomposition may be context dependent [32], [50], [51]. Given this complexity, the effect of amphibians on decomposition is likely influenced by the relative importance of top-down predatory effects on the invertebrate community and the bottom-up effects on available nutrients through ingestion and excretion [14]. For example, the top-down effects of red-backed salamanders on the invertebrate community are known to depend on the community composition and habitat heterogenity [50], [51], which suggests that the effects of salamanders on litter decomposition depends on the structural complexity of the environment and the biotic community. Litter decomposition is also likely influenced by the feeding behavior of microarthropods and their behavioral response to predation risk [40], [52]. For example, collembola, a primary prey item of red-backed salamanders, can decrease saprotrophic fungal biomass through direct grazing or increase fungal biomass by feeding preferentially on senescent fungal hyphae [53], [54]. Given the complex dynamics of forest floor food webs and the variable effect of red-backed salamanders on invertebrates [32], [51], [55], the effects of salamanders on ecosystem functions should be expected to be highly variable even when top-down effects predominate. The presence of earthworms can further complicate the relationship of salamanders and litter decomposition. Adult nightcrawlers (*Lumbricus terrestris*) burrows provide refuge for red-backed salamanders from extreme temperatures and predators, but reduce leaf litter and microarthropod abundance [56]. Juvenile nightcrawlers can be an important prey source for salamanders [56]. In small enclosures, the consumption of juvenile earthworms by adult salamanders reduced the rate of litter decomposition, but the presence of adult earthworms overwhelmed any effect of salamanders by breaking down nearly all leave litter [57]. Our upland, American beech-dominated sites had very few earthworms (*pers. obs.*), so the interactive effects of earthworms and salamanders in more natural settings remains to be tested.

Unlike litter, wood is decomposed almost entirely by saprotrophic fungi. Fungal activity is strongly influenced by temperature, moisture, and available nutrients, especially nitrogen [58]. Therefore, the pathways by which salamanders can influence woody decomposition are slightly more restricted than for litter decomposition. The potential ways salamanders can affect wood decomposition are still numerous and complex as they can derive from top-down effects on the food web or bottom-up effects on nutrient availability. As with litter decomposition, collembola can have variable effects on fungi and salamanders predatory effects on collembola populations may vary [35], [53], [55]. Therefore, it is difficult to predict the effect of salamanders on woody decomposition.

We did not find an effect of red-backed salamanders on birch dowel decomposition in either our large-scale depletion experiment or in our controlled, density enclosure experiment. There are a number of possible reasons for the lack of an observed effect. First, the top-down and bottom-up effects may roughly balance each other out or create sufficient variability to obscure salamander effects. Second, salamanders prey on wood-chewing invertebrates [18], [24], but these taxa may have been restricted from contact with the wood by the mesh sleeves around the dowels. Finally, wood decomposition is strongly influenced by nitrogen availability [58]; therefore, salamanders may have different effects depending on overall pools of available nitrogen. If salamanders make inorganic nitrogen more available for plants and fungi as suggested for abundant, terrestrial frogs [7], [11], [12], we would expect salamanders to have more influence on wood decomposition in systems with small pools of inorganic nitrogen.

On the contrary, our system had little available inorganic nitrogen and still no observable effect on wood decomposition. Unlike coqui frogs (*Eleutherodactylus coqui* [7]), salamanders may decrease nitrogen availability due to their slow turnover of nutrients, but this would have little effect on woody decomposition in a system already limited in nitrogen. Alternatively, the lack of salamander effect may be due to a limited microbial community structure for mineralizing nitrogen. We found relatively little net potential mineralization and virtually no net nitrification under idealized laboratory conditions. This suggests that nitrogen has already been immobilized by microbes and there is relatively little excess nitrogen available. However, soil N mineralization rates follow a periodic function [59] as does red-backed salamander activity [18]. It is possible that changes in available nitrogen from salamanders in the spring has a greater effect on N cycling and decomposition, since N mineralization remains low in the spring. Variability in salamander density may have obscured this effect in experiment 1 and we did not measure long-term decomposition or springtime decomposition in the better-controlled enclosure experiment.

We expected that salamanders would increase acorn germination through consumption of herbivores that may feed on germinating shoots as they pass through the leaf litter. We observed a trend of higher acorn germination in 2009 in the reference plots compared with the depletion plots. However, there was very little germination in the plots in 2010 or 2011 (Table 2). Low recruitment, likely due to dry, desiccating conditions in the week of germination in those years, may have obscured any effect of salamanders on germination. However, we did have high germination rates in most of the enclosures in 2011 despite the acorns being stratified from the same batch and planted within one week of acorns in reference plots. It is possible that the enclosures kept humidity and moisture levels slightly elevated, at least enough to maintain acorn viability through a mild spring drought. In the enclosures, we did not observe a significant effect of salamander density on germination. Red-backed salamanders might not consistently affect successful red oak germination in American beech-dominated forests depending on other environmental factors. Additionally, salamanders consume large numbers of invasive weevils (*Curculio spp.*) when available [60], which could affect germination in natural systems but was not evaluated in this study because we used only weevil-free acorns. Future studies would benefit from examining the effects of salamanders on recruitment of a variety of plant species in different forest types.

We also did not observe an effect of salamander density on rates of herbivory. However, we only measured foliar insect damage on red oak seedlings. There was very little foliar herbivory in general across density levels. However, lack of foliar damage to red oak seedlings may be limited to beech-dominated stands in southeastern New Hampshire. Homyack et al. [24] found that red-backed salamanders consumed numerous insect larvae, including fungus gnats (Order Diptera, Family Scaiariadae), which feed on plant tissue near the soil surface. Therefore, salamanders may influence herbivory of fine roots and mycorrhizal fungi through predation of fungus gnats and collembola. This could reduce plant growth and survival without generating differences in foliar herbivory. For example, more energy in the forest floor food web may be derived from belowground production, rather than decomposing detritus as previously thought [56]; therefore, salamanders could affect energy and nutrient flow in the system through effects on belowground herbivores and not foliar herbivores. Additionally, the effect would likely depend on the invertebrate community [35], [51] and possibly the plant species present.

In addition to ecosystem functions, we did not observe an effect of salamander depletions on the abundances of spiders, centipedes, or carabid beetles. This is in contrast to Hickerson et al. [26] who showed that red-backed salamander depletion resulted increased spider counts and decreased carabid beetle counts. Red-backed salamanders are known to act aggressively toward centipedes, and centipedes are less likely to be found under the same cover objects as salamanders [25] and that reduced centipede abundance can increase salamander counts under cover boards [26]. Additionally, red-backed salamanders are known to prey on spiders and other litter-dwelling invertebrate predators and spiders also prey on amphibians [18]. In the large-scale removal experiment, it is possible that variability in salamander densities obscured any potential effects of red-backed salamanders on these litter-dwelling invertebrate predators; however, in the enclosures the density of these predators did not co-vary with salamander density. There was also no evidence from our enclosures that the abundance of invertebrate predators affected the ecosystem functions. Homyack et al. [24] hypothesized that the lack of effect of red-backed salamanders on litter decomposition was a result of the effect of predatory invertebrates. However, between our two experiments we did not observe an effect of salamanders on predatory invertebrate abundance or an effect of predatory invertebrate abundance on ecosystem functions. This was surprising because spiders are known to reduce herbivore abundance and influence ecosystem functions [3], [5]. Spiders tend to reduce plant damage and increase plant biomass and reproduction [3]. However, this depends on the foraging tactics of the spiders. Active-hunting spiders increase primary production and N mineralization rates, whereas sit-and-wait spiders tend to have the opposite effect [5]. It is possible that both the food web structure and types of predatory invertebrate species present dampen ecosystem function effects of salamanders [6], [51]. Specifically, salamanders consume a broad range of invertebrates at difference trophic levels in the detrial food web. Thus, abundance of any single prey group may not be significantly reduced and top-down effects related to decomposition could be balanced by multi-trophic level feeding. In old fields, predator functional diversity effects on ecosystem functions can be linearly predicted from the individual effects [61]. Future studies interested in the effects of forest floor predators on ecosystem functions would benefit from explicitly manipulated densities of multiple predatory vertebrates and invertebrates in combination.

The complexity of the forest floor food web and mixture of top-down and bottom-up effects on ecosystem functions makes determining the effects of salamanders difficult, especially since the primary carbon source in forest floor food webs can be detrital or from roots [62]. In addition, there are experimental limitations that create further challenges in elucidating the effects of salamanders. There are always tradeoffs between realism, control, and replication when designing ecological experiments. Effects found in small, highly replicated, well-controlled experiments often do not extrapolate to more complex natural systems. In contrast, large-scale experiments have more realism but can lack the control and replication to detect the effects of specific manipulation.

In this study, we coupled these two approaches to examine the effects of red-backed salamanders on ecosystem functions. Logistics prevented precise determination of salamander abundance on all plots throughout the study. However, the cumulative evidence suggests significant reduction in salamander abundance on the depletion plots. It is possible that large variation among plots could have obscured the effects of depletions and that removals did not surface, subsurface, and age class portions of the population in proportion to their occurrence in the population. This is an inherent challenge with large-scale field manipulations, although we tried to minimize the ratio of between-plot to within-plot variability by using large plots that are variability in microhabitats. We did removal animals ranging in size from recent hatchlings to large adults, including gravid females, over multiple years, making it unlikely that this had a strong influence on the lack of observed effects by salamanders. Despite limitations associated with large-scale field manipulations, the combination of salamander depletions and controlled mesocosm enclosures provide insight into the role of red-backed salamanders in ecosystem functions. We did not find evidence of salamander impacts on decomposition, nitrogen cycling, foliar insect damage, or on predatory invertebrates. The inference from this study is limited to environmental conditions during the study in a beech-dominated forest in New Hampshire, although it may help our understanding of systems where predators influence ecosystem functions. Given complex interactions in soil food webs, the effects of habitat heterogeneity on top-down effects, and the mixture of top-down and bottom-up effects in forest ecosystems, it is likely that predator effects on ecosystem functions are context dependent. Future studies would benefit from additional controlled manipulations of the soil food web and predator densities when examining effects on ecosystem functions. Additionally, the plants and soil properties likely influence salamander effects and explicit study of salamander effects under different soil nutrient conditions and on different plant species would be informative for discerning context-dependent salamander effects. Finally, there may be significant time lags between changes in salamander densities and subsequent changes in ecosystem functions. Therefore, researchers should conduct studies over longer time periods in the future, especially in controlled mesocoms. We did find significant effects of year on ecosystem functions, likely due to natural variation in climatic conditions, and future studies over longer time periods could improve our understanding of this variation and determine if red-backed salamanders influence ecosystem functions under particular conditions.
